# Supplementary material for: Expression of IER3 in hepatocellular carcinoma: clinicopathology, prognosis, and potential regulatory pathways
Source: PeerJ. 2022 Mar 10;10:e12944. doi: 10.7717/peerj.12944 (PMC8918148; doi:10.7717/peerj.12944)
Supplement: Supplemental Information 1 — The expression of the IER3 protein was detected by in-house IHC in 94 cases of HCC tissues and in 127 cases of non-HCC liver tissues. The process and interpretation criteria of IHC were listed in the text material methods. IER3: Immediate early response 3; IHC: Immunohistochemistry; HCC: Hepatocellular carcinoma; AFP: Alpha fetoprotein. [file peerj-10-12944-s001.docx]

**Raw data of IER3 protein levels in HCC tissues assessed by in-house IHC**

| Tissue | Testing | IER3 protein score | Age | Gender | Pathologic grade | Alcohol history | Survival state | Survival time (months) | AFP (ng/mol) |
| --- | --- | --- | --- | --- | --- | --- | --- | --- | --- |
| HCC-1 | IER3 | 8 | 62 | MALE | I | YES | DECEASED | 2 | 4.15 |
| HCC-2 | IER3 | 8 | 53 | MALE | I | YES | DECEASED | 45 | 118.8 |
| HCC-3 | IER3 | 12 | 66 | MALE | I | YES | DECEASED | 10 | 4.67 |
| HCC-4 | IER3 | 12 | 62 | MALE | I~II | YES | DECEASED | 2 | 2.46 |
| HCC-5 | IER3 | 8 | 67 | MALE | I~II | YES | DECEASED | 20 | 151 |
| HCC-6 | IER3 | 12 | 39 | MALE | II | YES | DECEASED | 39 | 59.82 |
| HCC-7 | IER3 | 12 | 61 | MALE | II | YES | DECEASED | 23 | >1210 |
| HCC-8 | IER3 | 12 | 50 | MALE | II | YES | DECEASED | 3 | >1210 |
| HCC-9 | IER3 | 8 | 59 | MALE | II | YES | DECEASED | 5 | 5.69 |
| HCC-10 | IER3 | 12 | 59 | MALE | II | YES | DECEASED | 27 | 48.57 |
| HCC-11 | IER3 | 12 | 51 | MALE | II | YES | DECEASED | 16 | >1210 |
| HCC-12 | IER3 | 12 | 66 | MALE | II | YES | DECEASED | 84 | 717 |
| HCC-13 | IER3 | 12 | 43 | MALE | II | YES | DECEASED | 20 | 16778.4 |
| HCC-14 | IER3 | 12 | 73 | MALE | II | YES | DECEASED | 5 | 205.4 |
| HCC-15 | IER3 | 8 | 52 | MALE | II | YES | DECEASED | 37 | 207.1 |
| HCC-16 | IER3 | 12 | 38 | MALE | II | YES | DECEASED | 14 | >1210 |
| HCC-17 | IER3 | 12 | 31 | MALE | II | YES | DECEASED | 53 | >1210 |
| HCC-18 | IER3 | 12 | 57 | MALE | II | YES | DECEASED | 10 | >1210 |
| HCC-19 | IER3 | 12 | 47 | MALE | II | YES | DECEASED | 16 | >1210 |
| HCC-20 | IER3 | 12 | 41 | MALE | II | YES | DECEASED | 17 | >25410 |
| HCC-21 | IER3 | 12 | 50 | MALE | II | YES | DECEASED | 66 | 3.7 |
| HCC-22 | IER3 | 12 | 43 | MALE | II | YES | DECEASED | 30 | 8.93 |
| HCC-23 | IER3 | 12 | 60 | MALE | II | YES | DECEASED | 12 | >25410 |
| HCC-24 | IER3 | 12 | 58 | MALE | II | YES | DECEASED | 50 | >1210 |
| HCC-25 | IER3 | 8 | 57 | MALE | II | YES | DECEASED | 10 | >1210 |
| HCC-26 | IER3 | 12 | 61 | MALE | II | YES | DECEASED | 6 | 48.45 |
| HCC-27 | IER3 | 12 | 48 | MALE | II | YES | DECEASED | 14 | >1210 |
| HCC-28 | IER3 | 12 | 38 | MALE | III | YES | DECEASED | 49 | 419.5 |
| HCC-29 | IER3 | 12 | 55 | MALE | III | YES | DECEASED | 3 | 2.01 |
| HCC-30 | IER3 | 4 | 41 | MALE | III | YES | DECEASED | 20 | >1210 |
| HCC-31 | IER3 | 12 | 55 | MALE | III | YES | DECEASED | 32 | >1210 |
| HCC-32 | IER3 | 12 | 55 | MALE | III | YES | DECEASED | 5 | 24.16↑ |
| HCC-33 | IER3 | 8 | 52 | MALE | I-II | YES | DECEASED | 4 | 3.7 |
| HCC-34 | IER3 | 12 | 38 | MALE | I-II | YES | DECEASED | 43 | 2.38 |
| HCC-35 | IER3 | 8 | 32 | MALE | I-II | YES | DECEASED | 38 | >1210 |
| HCC-36 | IER3 | 8 | 58 | MALE | I-II | YES | DECEASED | 40 | 27.44 |
| HCC-37 | IER3 | 12 | 57 | MALE | I-II | YES | DECEASED | 45 | 5.69 |
| HCC-38 | IER3 | 12 | 43 | MALE | II-III | YES | DECEASED | 28 | 2.54 |
| HCC-39 | IER3 | 12 | 51 | MALE | II-III | YES | DECEASED | 8 | >1210 |
| HCC-40 | IER3 | 12 | 42 | MALE | II-III | YES | DECEASED | 16 | 2002 |
| HCC-41 | IER3 | 12 | 39 | MALE | II-III | YES | DECEASED | 25 | >49610 |
| HCC-42 | IER3 | 8 | 34 | MALE | II-III | YES | DECEASED | 40 | 91.89 |
| HCC-43 | IER3 | 12 | 58 | MALE | II-III | YES | DECEASED | 5 | 22.7 |
| HCC-44 | IER3 | 8 | 55 | MALE | - | YES | DECEASED | 50 | 138 |
| HCC-45 | IER3 | 8 | 52 | MALE | - | YES | DECEASED | 19 | 2.55 |
| HCC-46 | IER3 | 12 | 42 | MALE | - | YES | DECEASED | 47 | 4.1 |
| HCC-47 | IER3 | 12 | 44 | MALE | I~II | YES | LIVING | 105 | 6.37 |
| HCC-48 | IER3 | 12 | 50 | MALE | I~II | YES | LIVING | 106 | 68.18 |
| HCC-49 | IER3 | 12 | 53 | MALE | II | YES | LIVING | 116 | 128.5 |
| HCC-50 | IER3 | 8 | 54 | MALE | II-III | YES | LIVING | 72 | >1210 |
| HCC-51 | IER3 | 12 | 38 | MALE | II-III | YES | LIVING | 111 | 4.87 |
| HCC-52 | IER3 | 12 | 50 | FEMALE | I~II | NO | DECEASED | 46 | >1210 |
| HCC-53 | IER3 | 12 | 49 | FEMALE | II | NO | DECEASED | 18 | 1210 |
| HCC-54 | IER3 | 8 | 53 | FEMALE | II | NO | DECEASED | 102 | >1210 |
| HCC-55 | IER3 | 12 | 74 | FEMALE | II | NO | DECEASED | 6 | 4.37 |
| HCC-56 | IER3 | 12 | 42 | FEMALE | II | NO | DECEASED | 2 | >1210 |
| HCC-57 | IER3 | 12 | 69 | FEMALE | II | NO | DECEASED | 12 | 0.887 |
| HCC-58 | IER3 | 12 | 50 | FEMALE | II | NO | DECEASED | 13 | 229.40 |
| HCC-59 | IER3 | 12 | 42 | FEMALE | II | NO | DECEASED | 16 | >1210 |
| HCC-60 | IER3 | 8 | 45 | FEMALE | II | NO | DECEASED | 19 | 62.04 |
| HCC-61 | IER3 | 8 | 48 | MALE | II | NO | DECEASED | 11 | 18.6 |
| HCC-62 | IER3 | 12 | 73 | MALE | II | NO | DECEASED | 2 | (+) |
| HCC-63 | IER3 | 12 | 74 | MALE | II | NO | DECEASED | 3 | >1210 |
| HCC-64 | IER3 | 8 | 46 | MALE | II | NO | DECEASED | 19 | 9.11 |
| HCC-65 | IER3 | 8 | 49 | MALE | II | NO | DECEASED | 3 | 711.9 |
| HCC-66 | IER3 | 8 | 66 | MALE | II | NO | DECEASED | 11 | 86.76 |
| HCC-67 | IER3 | 8 | 38 | MALE | II | NO | DECEASED | 11 | 3.21 |
| HCC-68 | IER3 | 12 | 48 | MALE | II | NO | DECEASED | 29 | 18.6 |
| HCC-69 | IER3 | 12 | 80 | MALE | II | NO | DECEASED | 5 | 103.2 |
| HCC-70 | IER3 | 12 | 45 | MALE | II | NO | DECEASED | 37 | - |
| HCC-71 | IER3 | 8 | 58 | MALE | II | NO | DECEASED | 32 | 2.02 |
| HCC-72 | IER3 | 12 | 67 | MALE | II | NO | DECEASED | 6 | 15.02 |
| HCC-73 | IER3 | 12 | 50 | FEMALE | II~III | NO | DECEASED | 2 | 2.92 |
| HCC-74 | IER3 | 12 | 79 | FEMALE | III | NO | DECEASED | 30 | 13892.4 |
| HCC-75 | IER3 | 12 | 25 | FEMALE | III | NO | DECEASED | 25 | 2986.2 |
| HCC-76 | IER3 | 12 | 73 | FEMALE | III | NO | DECEASED | 3 | 2.93 |
| HCC-77 | IER3 | 12 | 40 | MALE | III | NO | DECEASED | 32 | 2053.7 |
| HCC-78 | IER3 | 12 | 41 | MALE | III | NO | DECEASED | 2 | 21.63 |
| HCC-79 | IER3 | 12 | 62 | FEMALE | I-II | NO | DECEASED | 2 | 61.79 |
| HCC-80 | IER3 | 12 | 50 | FEMALE | I-II | NO | DECEASED | 38 | >1210 |
| HCC-81 | IER3 | 8 | 44 | MALE | I-II | NO | DECEASED | 54 |  |
| HCC-82 | IER3 | 12 | 71 | MALE | I-II | NO | DECEASED | 14 | 441.8 |
| HCC-83 | IER3 | 12 | 61 | MALE | II-III | NO | DECEASED | 8 | 2.11 |
| HCC-84 | IER3 | 12 | 73 | MALE | II-III | NO | DECEASED | 7 | 864.2 |
| HCC-85 | IER3 | 8 | 48 | FEMALE | - | NO | DECEASED | 12 | - |
| HCC-86 | IER3 | 8 | 55 | FEMALE | - | NO | DECEASED | 15 | 1.79 |
| HCC-87 | IER3 | 12 | 71 | FEMALE | - | NO | DECEASED | 5 | 22.13 |
| HCC-88 | IER3 | 8 | 69 | FEMALE | - | NO | DECEASED | 1 | 0.808 |
| HCC-89 | IER3 | 12 | 40 | MALE | - | NO | DECEASED | 23 | 6.1 |
| HCC-90 | IER3 | 8 | 42 | MALE | - | NO | DECEASED | 8 | 2.36 |
| HCC-91 | IER3 | 8 | 69 | FEMALE | II | NO | LIVING | 102 | 39.61 |
| HCC-92 | IER3 | 12 | 46 | FEMALE | II | NO | LIVING | 108 | >1210 |
| HCC-93 | IER3 | 4 | 53 | MALE | III | NO | LIVING | 82 | 293.4 |
| HCC-94 | IER3 | 8 | 50 | FEMALE | I-II | NO | LIVING | 102 | 96.25 |
| Non-HCC-1 | IER3 | 4 | 52 | MALE | - | YES | - | - | 2 |
| Non-HCC-2 | IER3 | 8 | 55 | MALE | - | YES | - | - | 1.82 |
| Non-HCC-3 | IER3 | 4 | 41 | FEMALE | - | NO | - | - | 1.47 |
| Non-HCC-4 | IER3 | 0 | 43 | FEMALE | - | NO | - | - | 1.62 |
| Non-HCC-5 | IER3 | 4 | 39 | FEMALE | - | NO | - | - | 2.29 |
| Non-HCC-6 | IER3 | 8 | 55 | FEMALE | - | NO | - | - | 3.99 |
| Non-HCC-7 | IER3 | 8 | 51 | FEMALE | - | NO | - | - | 2.54 |
| Non-HCC-8 | IER3 | 2 | 36 | FEMALE | - | NO | - | - | 1.78 |
| Non-HCC-9 | IER3 | 8 | 56 | FEMALE | - | NO | - | - | 2.68 |
| Non-HCC-10 | IER3 | 8 | 42 | MALE | - | NO | - | - | 3.68 |
| Non-HCC-11 | IER3 | 8 | 55 | MALE | - | NO | - | - | 0.846 |
| Non-HCC-12 | IER3 | 4 | 33 | MALE | - | NO | - | - | 1.42 |
| Non-HCC-13 | IER3 | 4 | 45 | MALE | - | NO | - | - | 3.59 |
| Non-HCC-14 | IER3 | 8 | 39 | MALE | III | - | - | - | - |
| Non-HCC-15 | IER3 | 8 | 40 | MALE | II-III | - | - | - | - |
| Non-HCC-16 | IER3 | 8 | 49 | FEMALE | - | - | - | - | - |
| Non-HCC-17 | IER3 | 8 | 50 | FEMALE | - | - | - | - | - |
| Non-HCC-18 | IER3 | 4 | 53 | FEMALE | - | - | - | - | - |
| Non-HCC-19 | IER3 | 8 | 74 | FEMALE | - | - | - | - | - |
| Non-HCC-20 | IER3 | 8 | 79 | FEMALE | - | - | - | - | - |
| Non-HCC-21 | IER3 | 8 | 48 | FEMALE | - | - | - | - | - |
| Non-HCC-22 | IER3 | 12 | 42 | FEMALE | - | - | - | - | - |
| Non-HCC-23 | IER3 | 4 | 69 | FEMALE | - | - | - | - | - |
| Non-HCC-24 | IER3 | 8 | 50 | FEMALE | - | - | - | - | - |
| Non-HCC-25 | IER3 | 8 | 69 | FEMALE | - | - | - | - | - |
| Non-HCC-26 | IER3 | 12 | 62 | FEMALE | - | - | - | - | - |
| Non-HCC-27 | IER3 | 1 | 41 | FEMALE | - | - | - | - | - |
| Non-HCC-28 | IER3 | 4 | 43 | FEMALE | - | - | - | - | - |
| Non-HCC-29 | IER3 | 4 | 39 | FEMALE | - | - | - | - | - |
| Non-HCC-30 | IER3 | 8 | 50 | FEMALE | - | - | - | - | - |
| Non-HCC-31 | IER3 | 8 | 46 | FEMALE | - | - | - | - | - |
| Non-HCC-32 | IER3 | 12 | 55 | FEMALE | - | - | - | - | - |
| Non-HCC-33 | IER3 | 8 | 42 | FEMALE | - | - | - | - | - |
| Non-HCC-34 | IER3 | 4 | 71 | FEMALE | - | - | - | - | - |
| Non-HCC-35 | IER3 | 4 | 50 | FEMALE | - | - | - | - | - |
| Non-HCC-36 | IER3 | 8 | 45 | FEMALE | - | - | - | - | - |
| Non-HCC-37 | IER3 | 8 | 25 | FEMALE | - | - | - | - | - |
| Non-HCC-38 | IER3 | 8 | 50 | FEMALE | - | - | - | - | - |
| Non-HCC-39 | IER3 | 12 | 73 | FEMALE | - | - | - | - | - |
| Non-HCC-40 | IER3 | 4 | 69 | FEMALE | - | - | - | - | - |
| Non-HCC-41 | IER3 | 8 | 55 | FEMALE | - | - | - | - | - |
| Non-HCC-42 | IER3 | 8 | 51 | FEMALE | - | - | - | - | - |
| Non-HCC-43 | IER3 | 4 | 52 | FEMALE | - | - | - | - | 0.888 |
| Non-HCC-44 | IER3 | 1 | 52 | FEMALE | - | - | - | - | - |
| Non-HCC-45 | IER3 | 4 | 36 | FEMALE | - | - | - | - | - |
| Non-HCC-46 | IER3 | 4 | 52 | FEMALE | - | - | - | - | - |
| Non-HCC-47 | IER3 | 4 | 52 | FEMALE | - | - | - | - | - |
| Non-HCC-48 | IER3 | 2 | 56 | FEMALE | - | - | - | - | - |
| Non-HCC-49 | IER3 | 0 | 49 | FEMALE | - | - | - | - | - |
| Non-HCC-50 | IER3 | 1 | 49 | FEMALE | - | - | - | - | - |
| Non-HCC-51 | IER3 | 4 | 39 | MALE | - | - | - | - | - |
| Non-HCC-52 | IER3 | 8 | 61 | MALE | - | - | - | - | - |
| Non-HCC-53 | IER3 | 8 | 50 | MALE | - | - | - | - | - |
| Non-HCC-54 | IER3 | 8 | 62 | MALE | - | - | - | - | - |
| Non-HCC-55 | IER3 | 4 | 59 | MALE | - | - | - | - | - |
| Non-HCC-56 | IER3 | 4 | 48 | MALE | - | - | - | - | - |
| Non-HCC-57 | IER3 | 8 | 73 | MALE | - | - | - | - | - |
| Non-HCC-58 | IER3 | 4 | 52 | MALE | - | - | - | - | - |
| Non-HCC-59 | IER3 | 8 | 38 | MALE | - | - | - | - | - |
| Non-HCC-60 | IER3 | 4 | 54 | MALE | - | - | - | - | - |
| Non-HCC-61 | IER3 | 4 | 67 | MALE | - | - | - | - | - |
| Non-HCC-62 | IER3 | 8 | 74 | MALE | - | - | - | - | - |
| Non-HCC-63 | IER3 | 8 | 59 | MALE | - | - | - | - | - |
| Non-HCC-64 | IER3 | 8 | 62 | MALE | - | - | - | - | - |
| Non-HCC-65 | IER3 | 8 | 46 | MALE | - | - | - | - | - |
| Non-HCC-66 | IER3 | 8 | 49 | MALE | - | - | - | - | - |
| Non-HCC-67 | IER3 | 8 | 32 | MALE | - | - | - | - | - |
| Non-HCC-68 | IER3 | 8 | 51 | MALE | - | - | - | - | - |
| Non-HCC-69 | IER3 | 8 | 53 | MALE | - | - | - | - | - |
| Non-HCC-70 | IER3 | 8 | 55 | MALE | - | - | - | - | - |
| Non-HCC-71 | IER3 | 8 | 66 | MALE | - | - | - | - | - |
| Non-HCC-72 | IER3 | 8 | 43 | MALE | - | - | - | - | - |
| Non-HCC-73 | IER3 | 4 | 66 | MALE | - | - | - | - | - |
| Non-HCC-74 | IER3 | 8 | 38 | MALE | - | - | - | - | - |
| Non-HCC-75 | IER3 | 2 | 40 | MALE | - | - | - | - | - |
| Non-HCC-76 | IER3 | 8 | 73 | MALE | - | - | - | - | - |
| Non-HCC-77 | IER3 | 8 | 48 | MALE | - | - | - | - | - |
| Non-HCC-78 | IER3 | 4 | 61 | MALE | - | - | - | - | - |
| Non-HCC-79 | IER3 | 4 | 52 | MALE | - | - | - | - | - |
| Non-HCC-80 | IER3 | 8 | 38 | MALE | - | - | - | - | - |
| Non-HCC-81 | IER3 | 8 | 80 | MALE | - | - | - | - | - |
| Non-HCC-82 | IER3 | 8 | 31 | MALE | - | - | - | - | - |
| Non-HCC-83 | IER3 | 8 | 44 | MALE | - | - | - | - | - |
| Non-HCC-84 | IER3 | 8 | 53 | MALE | - | - | - | - | - |
| Non-HCC-85 | IER3 | 4 | 57 | MALE | - | - | - | - | - |
| Non-HCC-86 | IER3 | 4 | 58 | MALE | - | - | - | - | - |
| Non-HCC-87 | IER3 | 8 | 52 | MALE | - | - | - | - | - |
| Non-HCC-88 | IER3 | 4 | 42 | MALE | - | - | - | - | - |
| Non-HCC-89 | IER3 | 4 | 55 | MALE | - | - | - | - | - |
| Non-HCC-90 | IER3 | 4 | 33 | MALE | - | - | - | - | - |
| Non-HCC-91 | IER3 | 8 | 55 | MALE | - | - | - | - | - |
| Non-HCC-92 | IER3 | 4 | 43 | MALE | - | - | - | - | - |
| Non-HCC-93 | IER3 | 8 | 50 | MALE | - | - | - | - | - |
| Non-HCC-94 | IER3 | 8 | 38 | MALE | - | - | - | - | - |
| Non-HCC-95 | IER3 | 4 | 51 | MALE | - | - | - | - | - |
| Non-HCC-96 | IER3 | 4 | 55 | MALE | - | - | - | - | - |
| Non-HCC-97 | IER3 | 8 | 47 | MALE | - | - | - | - | - |
| Non-HCC-98 | IER3 | 8 | 45 | MALE | - | - | - | - | - |
| Non-HCC-99 | IER3 | 12 | 40 | MALE | - | - | - | - | - |
| Non-HCC-100 | IER3 | 8 | 52 | MALE | - | - | - | - | - |
| Non-HCC-101 | IER3 | 8 | 38 | MALE | - | - | - | - | - |
| Non-HCC-102 | IER3 | 8 | 41 | MALE | - | - | - | - | - |
| Non-HCC-103 | IER3 | 8 | 42 | MALE | - | - | - | - | - |
| Non-HCC-104 | IER3 | 4 | 55 | MALE | - | - | - | - | - |
| Non-HCC-105 | IER3 | 8 | 41 | MALE | - | - | - | - | - |
| Non-HCC-106 | IER3 | 6 | 53 | MALE | - | - | - | - | - |
| Non-HCC-107 | IER3 | 12 | 55 | MALE | - | - | - | - | - |
| Non-HCC-108 | IER3 | 12 | 42 | MALE | - | - | - | - | - |
| Non-HCC-109 | IER3 | 8 | 50 | MALE | - | - | - | - | - |
| Non-HCC-110 | IER3 | 12 | 43 | MALE | - | - | - | - | - |
| Non-HCC-111 | IER3 | 12 | 57 | MALE | - | - | - | - | - |
| Non-HCC-112 | IER3 | 4 | 60 | MALE | - | - | - | - | - |
| Non-HCC-113 | IER3 | 8 | 58 | MALE | - | - | - | - | - |
| Non-HCC-114 | IER3 | 4 | 57 | MALE | - | - | - | - | - |
| Non-HCC-115 | IER3 | 4 | 58 | MALE | - | - | - | - | - |
| Non-HCC-116 | IER3 | 4 | 67 | MALE | - | - | - | - | - |
| Non-HCC-117 | IER3 | 4 | 42 | MALE | - | - | - | - | - |
| Non-HCC-118 | IER3 | 8 | 34 | MALE | - | - | - | - | - |
| Non-HCC-119 | IER3 | 8 | 44 | MALE | - | - | - | - | - |
| Non-HCC-120 | IER3 | 8 | 61 | MALE | - | - | - | - | - |
| Non-HCC-121 | IER3 | 8 | 71 | MALE | - | - | - | - | - |
| Non-HCC-122 | IER3 | 4 | 41 | MALE | - | - | - | - | - |
| Non-HCC-123 | IER3 | 4 | 73 | MALE | - | - | - | - | - |
| Non-HCC-124 | IER3 | 8 | 48 | MALE | - | - | - | - | - |
| Non-HCC-125 | IER3 | 8 | 66 | MALE | - | - | - | - | - |
| Non-HCC-126 | IER3 | 8 | 58 | MALE | - | - | - | - | - |
| Non-HCC-127 | IER3 | 2 | 45 | MALE | - | - | - | - | - |

The expression of the IER3 protein was detected by in-house IHC in 94 cases of HCC tissues and in 127 cases of non-HCC liver tissues. The process and interpretation criteria of IHC were listed in the text material methods.

IER3: Immediate early response 3; IHC: Immunohistochemistry; HCC: Hepatocellular carcinoma; AFP: Alpha fetoprotein.
